# Lineage-specific determination of ring neuron circuitry in the central complex of *Drosophila*

**DOI:** 10.1242/bio.045062

**Published:** 2019-07-08

**Authors:** Jessika C. Bridi, Zoe N. Ludlow, Frank Hirth

**Affiliations:** Department of Basic and Clinical Neuroscience, Maurice Wohl Clinical Neuroscience Institute, Institute of Psychiatry, Psychology and Neuroscience, King's College London, London SE5 9RX, United Kingdom

**Keywords:** *Drosophila*, Brain, Stem cell lineage, Neural circuit, Ellipsoid body, Central complex

## Abstract

The ellipsoid body (EB) of the *Drosophila* central complex mediates sensorimotor integration and action selection for adaptive behaviours. Insights into its physiological function are steadily accumulating, however the developmental origin and genetic specification have remained largely elusive. Here we identify two stem cells in the embryonic neuroectoderm as precursor cells of neuronal progeny that establish EB circuits in the adult brain. Genetic tracing of embryonic neuroblasts ppd5 and mosaic analysis with a repressible cell marker identified lineage-related progeny as Pox neuro (Poxn)-expressing EB ring neurons, R1–R4. During embryonic brain development, *engrailed* function is required for the initial formation of Poxn-expressing ppd5-derived progeny. Postembryonic determination of R1–R4 identity depends on lineage-specific *Poxn* function that separates neuronal subtypes of ppd5-derived progeny into hemi-lineages with projections either terminating in the EB ring neuropil or the superior protocerebrum (SP). *Poxn* knockdown in ppd5-derived progeny results in identity transformation of engrailed-expressing hemi-lineages from SP to EB-specific circuits. In contrast, lineage-specific knockdown of *engrailed* leads to reduced numbers of Poxn-expressing ring neurons. These findings establish neuroblasts ppd5-derived ring neurons as lineage-related sister cells that require *engrailed* and *Poxn* function for the proper formation of EB circuitry in the adult central complex of *Drosophila*.

## Abstract (precis)

**Summary:** The formation of ring neurons is described from a pair of embryonic stem cells to *engrailed* and *Poxn neuro*-dependent lineage-specific determination of neural circuits in the central complex of *Drosophila*.

## INTRODUCTION

The *Drosophila* central complex is a composite of midline neuropils that include the protocerebral bridge, the fan-shaped body, the ellipsoid body (EB), the noduli and the lateral accessory lobes (Hanesch et al., 1989). These neuropils are interconnected in a modular way whereby columnar projection neurons leading to and from the central complex connect all its components that are themselves intersected by tangential layers of neural processes, which together form functional modules, each representing a segment of sensory space (Strausfeld, 2012). Functional studies have identified specific roles for the central complex in higher motor control, courtship and orientation behaviours, visual memory and place learning, as well as sleep, attention, arousal and decision-making (Strausfeld and Hirth, 2013; Pfeiffer and Homberg, 2014; Turner-Evans and Jayaraman, 2016).

In contrast to expanding insights into the physiological role of the central complex in regulating behaviour, its developmental origin and genetic specification has largely remained elusive. Earlier work described a primordial central complex at late larval/early pupal stages, which becomes fully formed by 48 h after puparium formation (Renn et al., 1999; Young and Armstrong, 2010). Genetic studies have identified several alleles of as-yet unidentified genes (Strauss and Heisenberg, 1993), as well as *orthodenticle* (Hirth et al., 1995), *Pax6/eyeless* (Callaerts et al., 2001), *Pox neuro* (*Poxn*) (Boll and Noll, 2002; Minocha et al., 2017), *tay-bridge* (Strauss and Heisenberg, 1993; Poeck et al., 2008), *roundabout* (Nicolas and Preat, 2005), *Pdm3* (Chen et al., 2012) and *semaphorin* (Xie et al., 2017) as genes involved in normal formation of central complex sub-structures (for review see Furukubo-Tokunaga et al., 2012; Strausfeld and Hirth, 2013).

Here we investigate the origin and formation of EB ring neurons R1–R4 in the developing and adult brain of *Drosophila*. We identify bilateral symmetric neuroblasts ppd5 in the embryonic procephalic neuroectoderm as founder cells of neuronal progeny that constitute R1–R4 subtypes of tangential ring neurons in the adult EB. Mutant analysis and targeted genetic manipulations reveal a lineage-specific requirement of *engrailed* (*en*) and *Poxn* activity that determines the number and identity of ppd5-derived progeny and their EB ring-specific connectivity pattern in the adult central complex of *Drosophila*.

## RESULTS

### EB ring neurons are lineage-derived progeny of embryonic neuroblasts ppd5

**Fig. 1.**
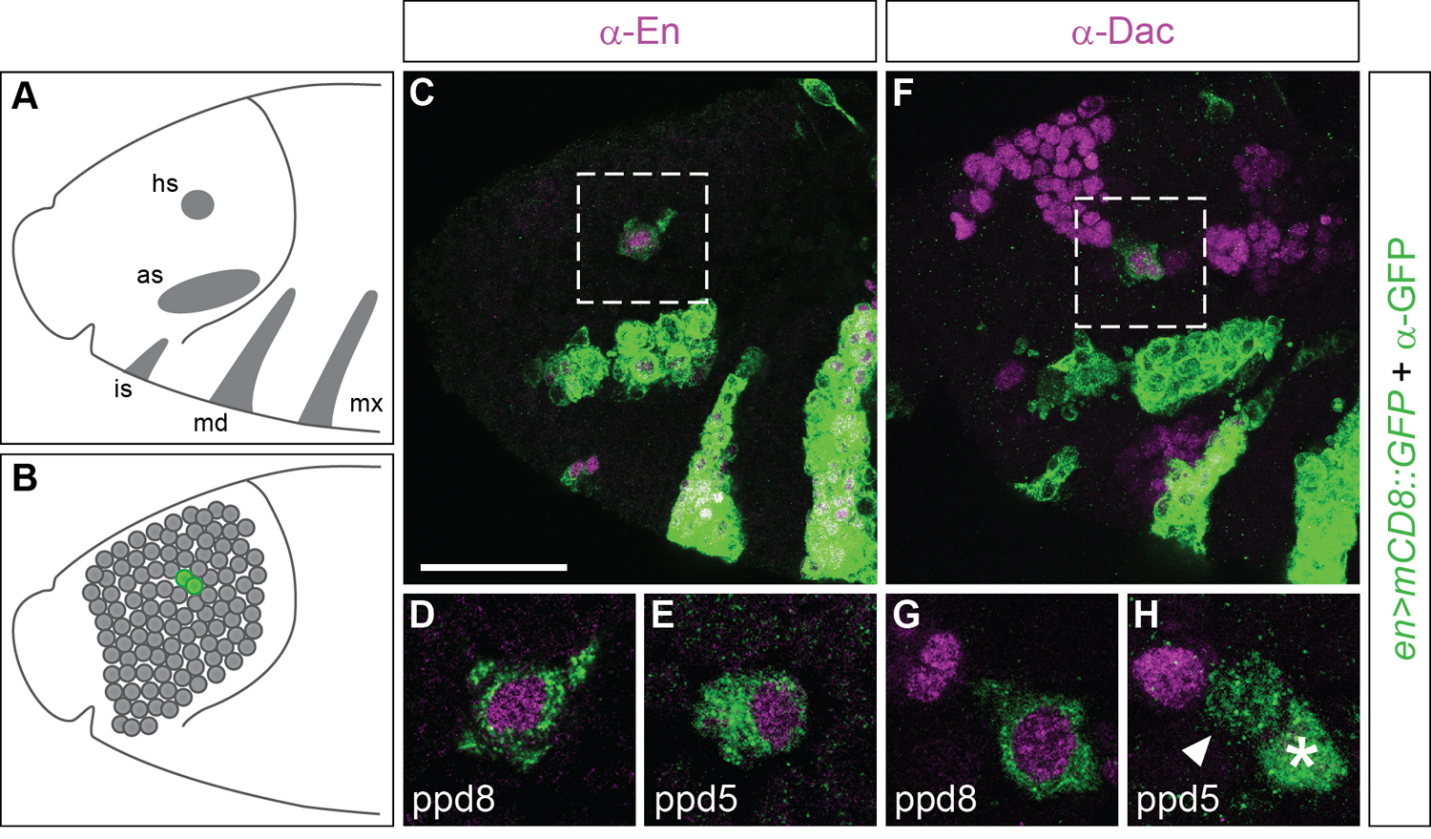
**Engrailed-expressing embryonic neuroblasts ppd5 and ppd8.** (A) Schematic of stage 11 embryo showing Engrailed (En) expression in the ectoderm (grey areas: hs, head spot; as, antennal stripe; is, intercalary stripe; md, mandibular stripe; mx, maxillary stripe) and (B) in the neuro-ectoderm from which brain neuroblasts delaminate (B, grey dots); these include neuroblasts ppd5 and ppd8 (B, green dots) that derive from the En head spot. Lateral views, anterior to the left. (C–H) At stage 11, *en>mCD8::GFP* (green) visualises expression patterns that mimic endogenous En expression, including the head spot (C,F, dashed areas) as well as neuroblasts ppd8 (D) and ppd5 (E) that both express *mCD8::GFP* (green) and En (magenta). (F) Dachshund (Dac, magenta) expression in the anterior head ectoderm is also found in the En head spot (F, dashed area) and in neuroblast ppd8 (G) but not in neuroblast ppd5 (H, arrowhead), both of which express *en>mCD8::GFP* (in H, ppd8 is highlighted with asterisk)*.* D and E are enlargements of the dashed area in C at different focal planes; G and H are enlargements of the dashed area in F at different focal planes. C,F, projections of confocal sections; D,E,G, single sections; H, two confocal sections. *n*>20 for each condition. Scale bar: 25 μm.

**Fig. 2.**
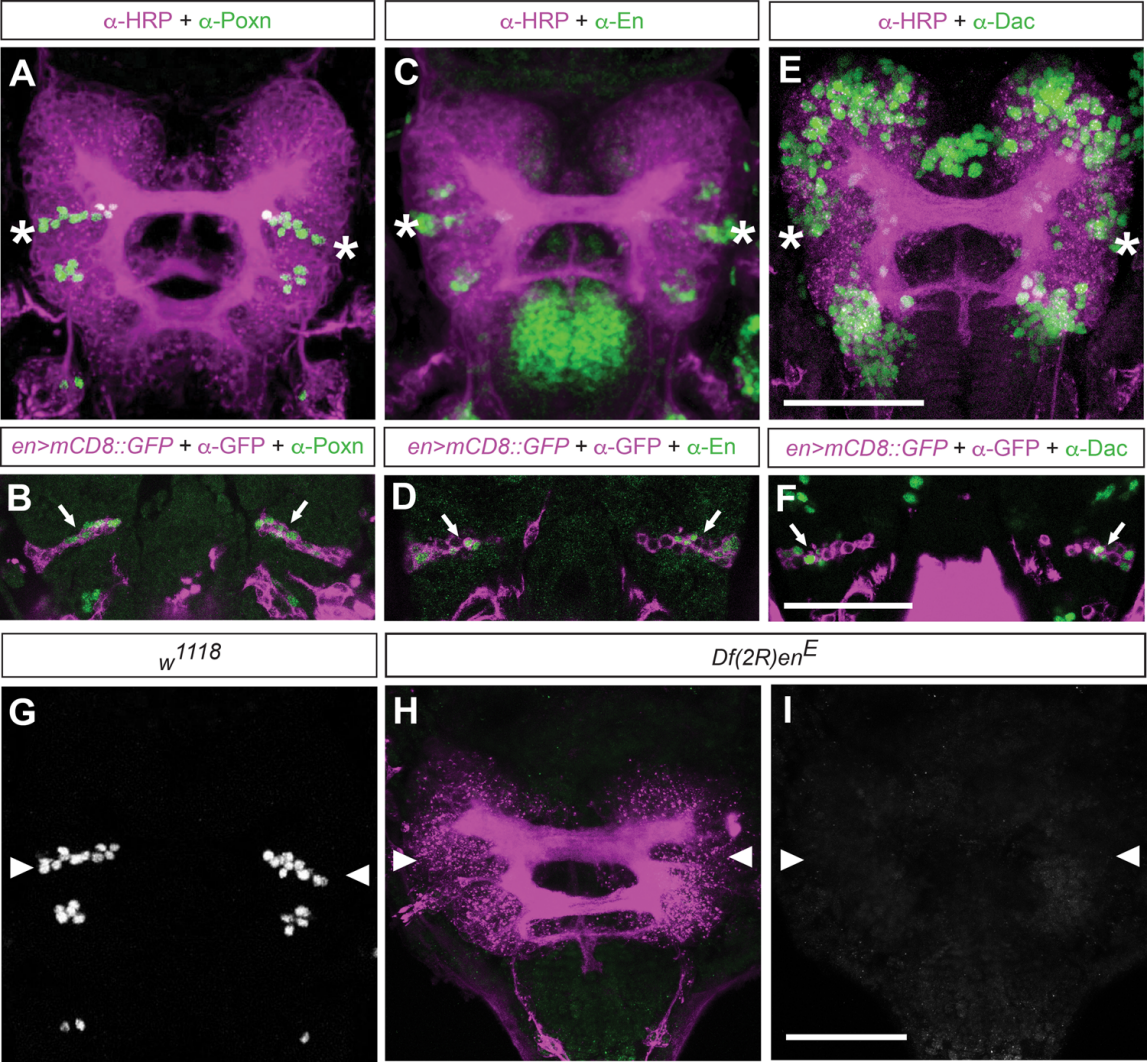
**Neuroblasts ppd5/8-derived neural lineages in the embryonic brain express Poxn and require *engrailed* function.** (A,C,E,G) Stage 14 embryonic *w^1118^* control brains labelled with anti-HRP (A,C,E,magenta). Dorsal views, anterior up. (B,D,F) *en-Gal4*-driven *UAS-mCD8::GFP* expression visualises progeny of neuroblasts ppd5/8 in the posterior protocerebrum (magenta), single confocal sections. (A,G) Poxn expression (green) is detectable in two clusters in the brain (HRP, magenta), in the protocerebrum (asterisks) and deutocerebrum; note that A and G show the same brain. (B) anti-Poxn immunolabelling (green) identifies Poxn expression in *en>mCD8::GFP*-labelled ppd5/8 progeny (white arrows). (C) En expression (green) can be seen in three clusters in the embryonic brain; in the anterior protocerebrum, posterior protocerebrum (asterisks) and posterior deutocerebrum. (D) Anti-En immunolabelling (green) identifies En expression in *en>mCD8::GFP*-labelled ppd5/8 progeny (white arrows). (E) Dachshund (Dac, green) is expressed in several areas of the embryonic brain including the posterior protocerebrum (asterisks). (F) Anti-Dac immunolabelling (green) identifies Dac expression in *en>mCD8::GFP*-labelled ppd5/8 progeny (white arrows). (G) Single-channel image of A showing Poxn-expressing cells, including posterior protocerebral cluster (arrowheads). (H) Embryonic brain of homozygous deficiency *Df(2R)en^E^*-labelled with anti-HRP (magenta); arrowheads indicate the position of the posterior protocerebrum, which is devoid of Poxn immunolabelling. (I) Single channel showing absence of Poxn expression in the brain. *n*>20 for each condition. Scale bars: 50 μm.

**Fig. 3.**
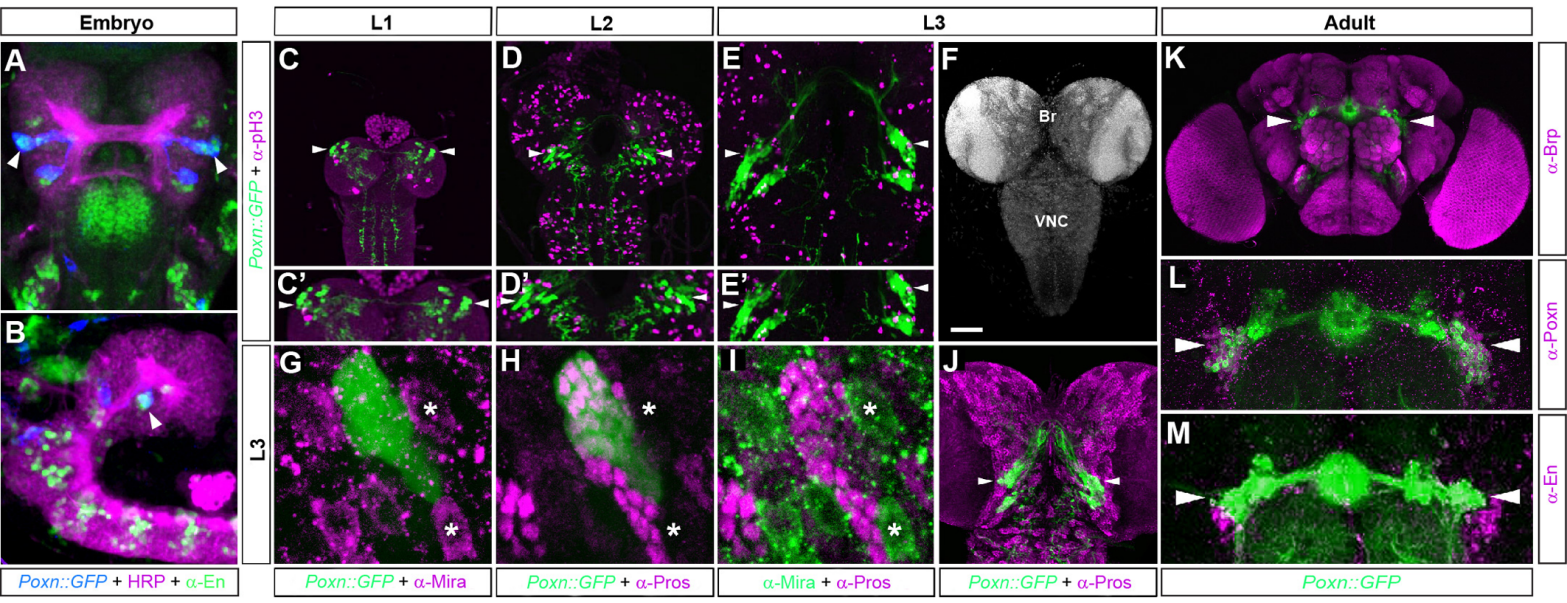
**EB precursor cell expansion and axogenesis of *Poxn::GFP*-labelled ring neurons.** (A–M) *Poxn-Gal4>UAS-mCD8::GFP*-labelled brains visualise GFP-expressing neurons in the posterior protocerebrum of the embryonic brain at stage 14 (A,B), during larval stages L1 (C,C′), L2 (D,D′), L3 (F–J) and in the adult (K–M). (A,B) *Poxn>mCD8::GFP* expression visualises protocerebral Poxn+ lineages (blue) in the embryonic brain (anti-HRP, magenta); co-immunolabelling with anti-Engrailed (green) reveals that embryonic Poxn-Gal4+ cells co-express Engrailed (arrowheads; A, frontal view; B, lateral view). (C–E′) Immunolabelling with anti-pH3 (magenta) visualises phosphorylated Histone H3 as a marker of mitotic activity. (G–J) Immunolabelling with anti-Miranda (anti-Mira) visualises precursor cells (G,I, asterisks) and reveals that *Poxn>mCD8::GFP* cells are devoid of Miranda expression. Immunolabelling with anti-Prospero (anti-Pros) labels differentiating neurons (H–J) in the larval brain (Br) and ventral nerve cord (VNC); posterior protocerebral *Poxn>GFP* cells co-express Prospero and during larval stages L2/L3 send neuronal projections towards the midline (J, arrowheads). *Poxn>mCD8::GFP* visualises adult EB ring neurons (K–M, arrowheads) that are immunoreactive for anti-Poxn (L) but not for anti-Engrailed, which labels cells adjacent to *Poxn>mCD8::GFP*-positive ring neurons (M). *n*>20 for each condition. Scale bar: 50 μm.

To gain insights into the origin and formation of the EB, we followed the expression of the Pax2/5/8 homologue *Poxn* which is expressed in the developing and adult EB as revealed by full enhancer analysis (Boll and Noll, 2002). In the embryonic protocerebrum, Poxn expression can be found at the protocerebral/deutocerebral neuromere boundary, which is also characterised by Engrailed-expressing cells (Hirth et al., 2003). These Engrailed-expressing cells derive from neuroblasts ppd5 and ppd8 (Urbach and Technau, 2003; Urbach et al., 2003), which are distinguishable by dachshund (Dac) expression that is restricted to ppd8. Ppd5/8 neuroblasts can be visualised with *en-Gal4* (Kumar et al., 2009) when combined with *UAS-mCD8::GFP* expression (Fig. 1), which reveals that neuroblasts ppd5/8 form bilaterally-symmetric lineages in the embryonic brain. The resulting neural progeny of ppd5/8 start to express *Poxn*, which can be visualised with *en>mCD8::GFP* (Fig. 2A–D) but also with *Poxn>mCD8::GFP*, which reveals that Poxn-Gal4+ cells in the embryonic brain are labelled by Engrailed (Fig. 3A,B, arrowheads). During larval development, Poxn expression is maintained in these lineages as demonstrated by *Poxn>mCD8::GFP* (Fig. 3C–I) and anti-Poxn immunolabelling (Fig. S1). By larval stage late-L2/early-L3, *Poxn>mCD8::GFP*-labelled neurons can be identified that send projections towards the midline of the central brain (Fig. 3E,J). In the adult brain, *Poxn>mCD8::GFP* labels EB ring neurons (Fig. 3K–M, arrowheads) that no longer express Engrailed (Fig. 3M). Together these data suggest that Poxn-expressing EB ring neurons might be clonally related progeny of *en*-expressing neuroblasts ppd5.

**Fig. 4.**
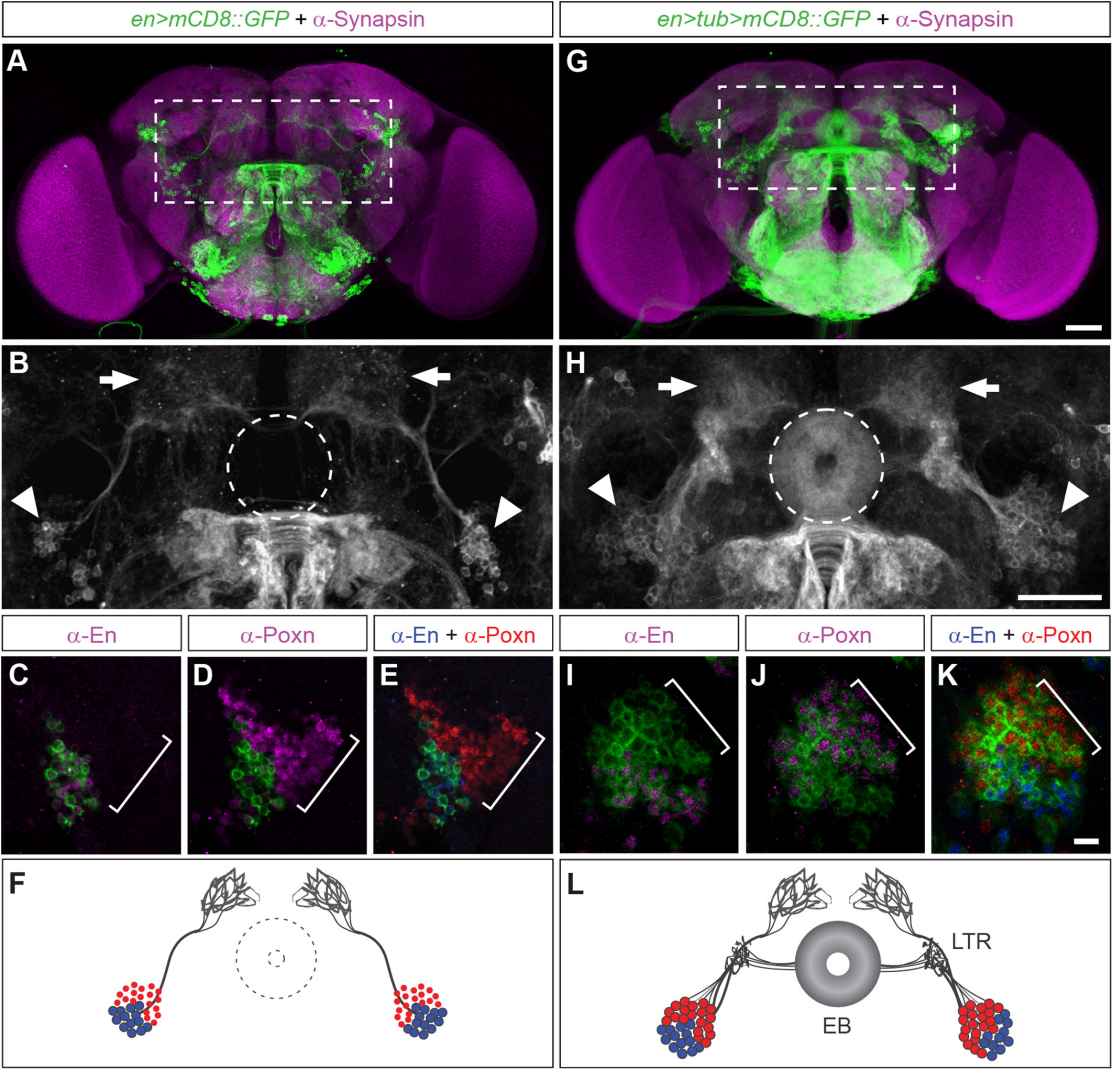
**Genetic tracing of engrailed-expressing ppd5** **lineages reveals Poxn-expressing EB ring neurons in the adult brain.** (A) *en>mCD8::GFP* expression in the adult brain (dashed area enlarged in B). (B) *en>mCD8::GFP*-expressing cells (arrowheads) in the posterior protocerebrum; they project towards and arborise the SP (arrows) dorsal to the EB neuropil (outlined as dashed circle). (C–E) *en>mCD8::GFP*-labelled cells (square bracket) located in the posterior protocerebrum express Engrailed (C) but not Poxn (D), showing no overlap of anti-En and anti-Poxn (E). (F) Schematic of *en>mCD8::GFP*-targeted cells and their projections in the adult brain: En-expressing cells (blue) reside adjacent to Poxn-expressing cells (red) and send axons (grey) to the SP. (G) Genetic tracing of *en>tub>mCD8::GFP* in the adult brain (dashed area enlarged in H) visualises cells and axons similar to, but stronger than *en>mCD8::GFP* (compare to A) as well as ring neurons and the EB neuropil. (H) *en>tub>mCD8::GFP*-expressing cells (arrowheads) in the posterior protocerebrum project towards and arborise into the SP (arrows) but also into the ellipsoid neuropil (dashed circle). (I–K) *en>tub>mCD8::GFP*-expressing cells (square bracket) located in the posterior protocerebrum express En (I) and Poxn (J). Note that anti-En does not overlap with anti-Poxn immunoreactivity (K). (L) Schematic of *en>tub>mCD8::GFP* targeted cells and their projections in the adult brain; En-expressing cells (blue) reside adjacent to Poxn-expressing cells (red) and send projections (grey) to the SP; Poxn-expressing neurons (red) send projections (grey) along the lateral triangle (LTR) into the EB ring neuropil (EB). *n*>20 for each condition. Scale bars: 50 μm in G,H; 10 μm in K.

To test this hypothesis, we used a combination of Gal4/UAS and FLP/FRT cassettes (Roy et al., 2007) allowing the inheritance of a traceable, membrane-tethered marker (*mCD8::GFP*) which identifies progeny that share a common origin and are therefore clonally related. We first utilised the *en-Gal4* driver line with Gal4 expression detectable from early embryogenesis in the procephalic neuroepithelium (Fig. 1) and that remains active throughout development and in the adult (Fig. S2). Analysis of *en>mCD8::GFP* flies co-labelled with anti-En revealed expression of endogenous Engrailed always within *mCD8::GFP-*labelled cells, including neuroblasts ppd5 (Fig. 1C–E) and their progeny in the embryonic (Fig. 2C,D), larval (Fig. S2A–E) and adult brain (Fig. 4A–F and Fig. S2F–I). These data establish that *en>mCD8::GFP* recapitulates the spatio-temporal pattern of endogenous *engrailed* expression.

Anatomical and immunohistochemical analysis of *en>mCD8::GFP* brains revealed that GFP-labelled En-expressing cells extend projections during larval development towards the midline of the central brain (Fig. S2A,B), which in the adult brain of *en>mCD8::GFP* flies terminate in the superior protocerebrum (SP) (Fig. S2F). In addition to cell-specific labelling of *en>mCD8::GFP* projection patterns, cell- and lineage-specific labelling using *en*-mediated activation of the constitutively active tubulin driver *tub-Gal4* (*en>tub>mCD8::GFP*) (Fig. S3) invariably visualised the EB neuropil and EB-specific ring neurons (Fig. 4G–L and Fig. S4; *n*=77 brains). Labelling of *en>tub>mCD8::GFP*-expressing cells in the posterior protocerebrum revealed neuronal projections that terminate in the SP (Fig. 4H, arrows) as well as in the ellipsoid neuropil (Fig. 4H, arrowheads). Labelling *en>mCD8::GFP* brains with anti-Poxn showed hardly any overlap between GFP and Poxn expression (Fig. 4C–E), which is detectable immediately adjacent to En-expressing cells (Fig. 4E). However, *en>tub>mCD8::GFP* brains immunolabelled with anti-Poxn revealed that protocerebral Poxn-expressing cells were co-labelled with *mCD8::GFP* and were located immediately adjacent to cells expressing GFP and En (Fig. 4I–K, compare to C to E). These data suggest that Poxn-expressing neurons labelled with *en>tub>mCD8::GFP* share a common lineage relationship with Engrailed-expressing cells.

To corroborate these findings, we carried out mosaic analysis with a repressible cell marker (MARCM) (Lee and Luo, 1999) utilising a tubulin-Gal4 driver. Neuroblast lineage labelling was induced in early L1 and adult brains were screened for GFP expression in both Engrailed-expressing cells projecting to the SP and Poxn-expressing cells projecting to the EB. Following this protocol, we identified Engrailed and Poxn-expressing MARCM-labelled cells, both of which initially project together anterior-medially, before Engrailed-expressing cells branch off to the SP and Poxn-expressing cells project to the EB ring neuropil (Fig. S5). MARCM thus demonstrates that Engrailed and Poxn-expressing cells in the posterior protocerebrum are clonally related. Together with lineage tracing using *en>tub>mCD8::GFP*, our findings identify Poxn-expressing EB ring neurons and neighbouring SP-projecting Engrailed-expressing cells as clonally-related progeny that constitute two hemi-lineages derived from Engrailed-expressing neuroblasts ppd5.

### ppd5 neuroblast-derived progeny form part of EB R1-R4 ring neuron circuitry

We next wanted to know to which ring-neuron subtypes these Poxn-expressing EB-precursor cells give rise. Adult EB neurons are classified as large-field ring neurons based on their subtype-specific stereotypical pattern of synapse formation (Hanesch et al., 1989; Renn et al., 1999; Young and Armstrong, 2010). Previous reports identified and visualised R1–R4 neurons using subtype-specific Gal4 driver lines (Renn et al., 1999; Wang et al., 2002; Martín-Peña et al., 2006; Young and Armstrong, 2010; Shaw et al., 2018), which combined with *mCD8::GFP*, reveal that axon terminals of R1–R3 neurons enter via the EB canal and synapse outwardly at different positions within the EB ring, whereas R4 projections reach the EB at the distal surface and synapse in the outer ring (Fig. S6). We made use of these Gal4 lines to investigate whether Poxn-expressing cells comprise different EB ring-neuron subclasses.

In the adult brain, Poxn expression is detectable in GFP-labelled ring neurons (Fig. S6) of *c105>mCD8::GFP*, *c819>mCD8::GFP*, and *c507>mCD8::GFP*. We also tested other Gal4 strains, including *EB1-Gal4* (Wang et al., 2002), *c232-Gal4* and *c42-Gal4* (Renn et al., 1999) as well as *796-Gal4* (Martín-Peña et al., 2006), that label ring-neuron subtypes partially overlapping with c105, c819 and c507. In all cases examined, we detected anti-Poxn immunoreactivity in nuclei of *mCD8::GFP*-labelled cells (Fig. S6B–H), which altogether demonstrates that Poxn expression can be found in ring-neuron subtypes R1–R4. These data suggest Engrailed-expressing neuroblasts ppd5 give rise to Poxn expressing progeny that comprise ring-neuron subtypes R1–R4 of EB-specific circuitry.

### Embryonic formation of Poxn-expressing lineages requires *engrailed* function

Our lineage analysis identified Poxn-expressing ring neurons as progeny of Engrailed expressing neuroblasts ppd5, suggesting that *engrailed* might be required for their development and/or specification. To investigate these hypotheses, we first analysed two different alleles affecting *engrailed* function. *en^CX1^* affects embryonic patterning but does not completely remove the *engrailed* orthologues *en* and *invected* (*inv*) (Heemskerk et al., 1991). *Df(2R)en^E^* is a deficiency removing the entire *en* locus and the majority of the *inv* locus, resulting in the absence of *en* and *inv* gene products, which is therefore considered to be a null allele of *engrailed* (Tabata et al., 1995).

Analysis of the embryonic brain and ventral nervous system of *Df(2R)en^E^*-homozygous mutants revealed severe patterning defects including absent or fused commissures, fused or broken connectives and a disrupted peripheral nerve pattern. Anti-Poxn immunolabelling of these mutant brains revealed a complete absence of Poxn-labelled neurons in 94.7% (*n*=19) of all cases examined that developed beyond stage 13 (Fig. 2H,I). These data suggest that *engrailed* is required for the formation of Poxn-expressing progeny in the embryonic protocerebrum.

### Determination of ring-neuron identity depends on lineage-specific *Poxn* function

**Fig. 5.**
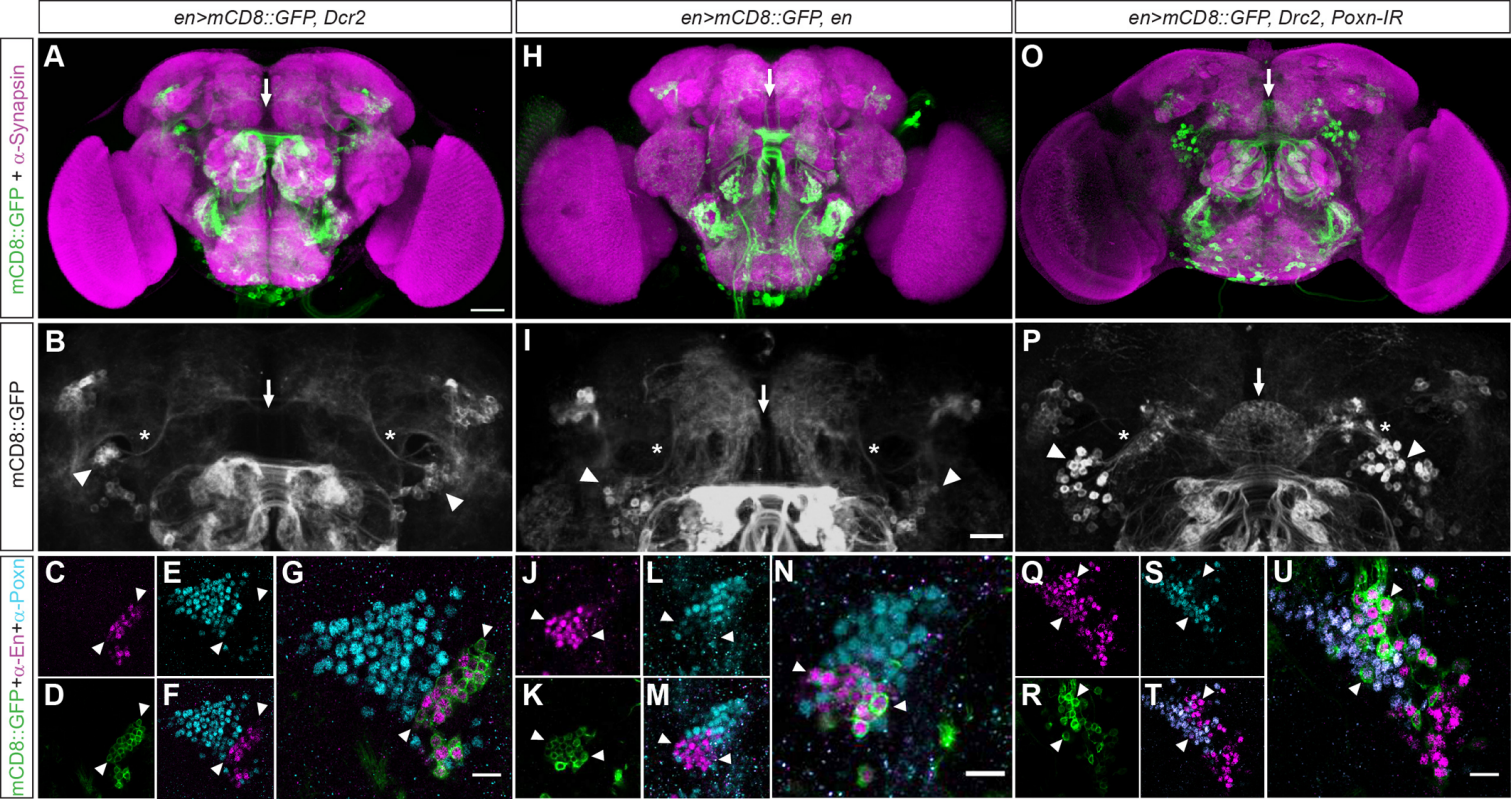
**Lineage-specific genetic manipulation of *engrailed* and *Poxn*.** Confocal images of whole-mount adult brains expressing *mCD8::GFP* and immunolabelled either with anti-Syntaxin/3C11 (A,H,O, magenta) or with anti-Engrailed (C,F,G,J,M,N,Q,T,U, magenta) and anti-Poxn (E,F,G,L,M,N,S,T,U, cyan). Dorsal is up. (A) In *en>mCD8::GFP,Dcr2* the EB neuropil is not visualised (arrows). (B) GFP-expressing cells (arrowheads) send their projections ipsilaterally (asterisks) to the superior protocerebrum (SP), dorsal to EB neuropil (arrow). (C) *en>mCD8::GFP* cells express engrailed and (D) GFP, but (E) not Poxn, which together are (F) expressed in close vicinity but (G) non-overlapping (arrowheads). (H) In *en>mCD8::GFP,en,* UAS-mediated overexpression of *engrailed* reveals (I) GFP-expressing cells (arrowheads) sending projections ipsilaterally (asterisks) to the SP (arrow indicates position of EB neuropil). (J) *en>mCD8::GFP,en* cells express engrailed and (K) GFP, but (L) not Poxn, which together are (M) expressed in close vicinity but (N) non-overlapping (arrowheads); compare to corresponding control in Fig. 4A–E. (O) In *en>mCD8::GFP, Dcr2, Poxn-IR*, RNAi-mediated knockdown of *Poxn* in ppd5/8 lineages reveals GFP-positive EB neuropil (arrow); (P) GFP-expressing cells (arrowheads) send their projections contralaterally (asterisks) into the EB neuropil (arrow). (Q) *en>mCD8::GFP, Dcr2, Poxn-IR* cells express engrailed and (R) GFP, some of which (S) also express Poxn, which together (T) are co-expressed in (U) some GFP-expressing ring neurons (arrowheads). *n*>10 for each condition. Scale bars: 50 μm in A; 10 μm in G,N,U.

The extended post-embryonic phase of EB lineage development made it necessary to bypass embryonic lethality associated with recessive lethal mutations, as seen for *Df(2R)en^E^* homozygous mutants. Moreover, previous studies had shown that *Poxn* mutants are adult viable but present with an affected EB neuropil (Boll and Noll, 2002; Minocha et al., 2017). We therefore used lineage-specific genetic manipulations to gain insights into the mechanisms of *engrailed-* and *Poxn*-mediated EB development. To this end, we used UAS-mediated overexpression and RNA interference (RNAi) targeted by *en-Gal4* and co-expressed *Dicer-2* (*Dcr2*) to enhance RNAi efficiency (Dietzl et al., 2007). We first tested whether on its own, *en-Gal4*-mediated *UAS-Dcr2* expression interfered with lineage formation and EB development. For this we analysed adult brains of *en>mCD8::GFP* controls and *en>mCD8::GFP,Dcr2* co-immunolabelled with anti-Poxn to visualise Poxn-expressing ring neurons, and with anti-En to visualise adjacent hemi-lineage neurons projecting to the SP. These data revealed indistinguishable patterns of Poxn and Engrailed expression in the brains of both genotypes (Fig. 5A–G, compare to Fig. 4A–E and Fig. S7A–F), suggesting that *en-Gal4*-driven ectopic activation of *Dcr2* does not affect neuroblast ppd5 lineage formation and EB development.

We next studied whether overexpression of *engrailed* and *Poxn* might interfere with lineage formation and EB development. Analysis of *en>mCD8::GFP,en* brains revealed projection patterns and anti-Poxn and anti-Engrailed immunolabelling (Fig. 5H–N) indistinguishable from controls (Fig. 4A–E). In contrast, we were not able to analyse adult brains of *en-Gal4*-mediated, lineage-specific overexpression of *UAS-Poxn* due to early developmental lethality of *en>mCD8::GFP,Poxn* flies. We then analysed the brain phenotypes of RNAi-mediated knockdown of *engrailed* and *Poxn*. Again, we were not able to analyse adult brains of *en>mCD8::GFP,Dcr2,en-IR* animals due to early developmental lethality.

In contrast, RNAi-mediated knockdown of *Poxn* (*en>mCD8::GFP,Dcr2,Poxn-IR*) revealed a striking EB phenotype (Fig. 5O–U). Although *en-Gal4* normally does not target EB ring neurons in the adult brain (Figs 4A–E, 5A-G and Fig. S7A–F), we observed GFP-expressing cells projecting to the EB in *en>mCD8::GFP, Dcr2, Poxn-IR* brains (Fig. 5O,P and Fig. S7G-L). These *en>mCD8::GFP,Dcr2,Poxn-IR*-expressing neurons revealed the typical morphology and projection pattern of tangential ring neurons, in that they send axons via the lateral triangles to terminate in the EB neuropil. Immunohistochemical analysis detected Poxn-expression clusters, however GFP expression was also seen in cells immunolabelled with anti-Poxn (Fig. 5Q–U) despite the fact that the utilised *UAS-Poxn-IR* led to knockdown of *Poxn* to levels undetectable by immunohistochemistry (Fig. S8). Notably, anti-En immunostaining identified GFP-labelled EB ring neurons that express both Engrailed and Poxn (Fig. 5U, compare with E–G), which is normally never seen for *en-Gal4*-labelled adult neurons typically projecting to the SP (see Fig. 4A–E), nor for Poxn-expressing ring neurons that usually do not co-express Engrailed (Figs 4I–K and 5E–G). Furthermore, in *en>mCD8::GFP,Dcr2,Poxn-IR* brains we could not detect GFP-labelled cells projecting to the SP (Fig. 5O,P and Fig. S8G–L) that are normally seen in *en>mCD8::GFP* brains (Fig. 4A,B, arrows), in related *en>mCD8::GFP,Dcr2* controls (Fig. 5A,B), and also detectable in genetically traced *en>tub>mCD8::GFP* brains (Fig. 4G,H, arrows). Instead, GFP-labelling of *en>mCD8::GFP,Dcr2,poxn-IR* brains frequently revealed a ventrally open EB ring neuropil (Fig. S7G–L), devoid of the toroidal ring shape that is normally seen in *Poxn::GFP* and genetically traced *en>tub>mCD8::GFP* brains (Fig. 4G,H). These findings suggest that *en-Gal4*-mediated knockdown of *Poxn* transforms the identity of ppd5-derived hemi-lineages from Engrailed-expressing SP-projecting neurons to ring neurons that send terminal projections to the EB neuropil.

### Specification of ring-neuron number requires *Poxn* and *engrailed* function

**Fig. 6.**
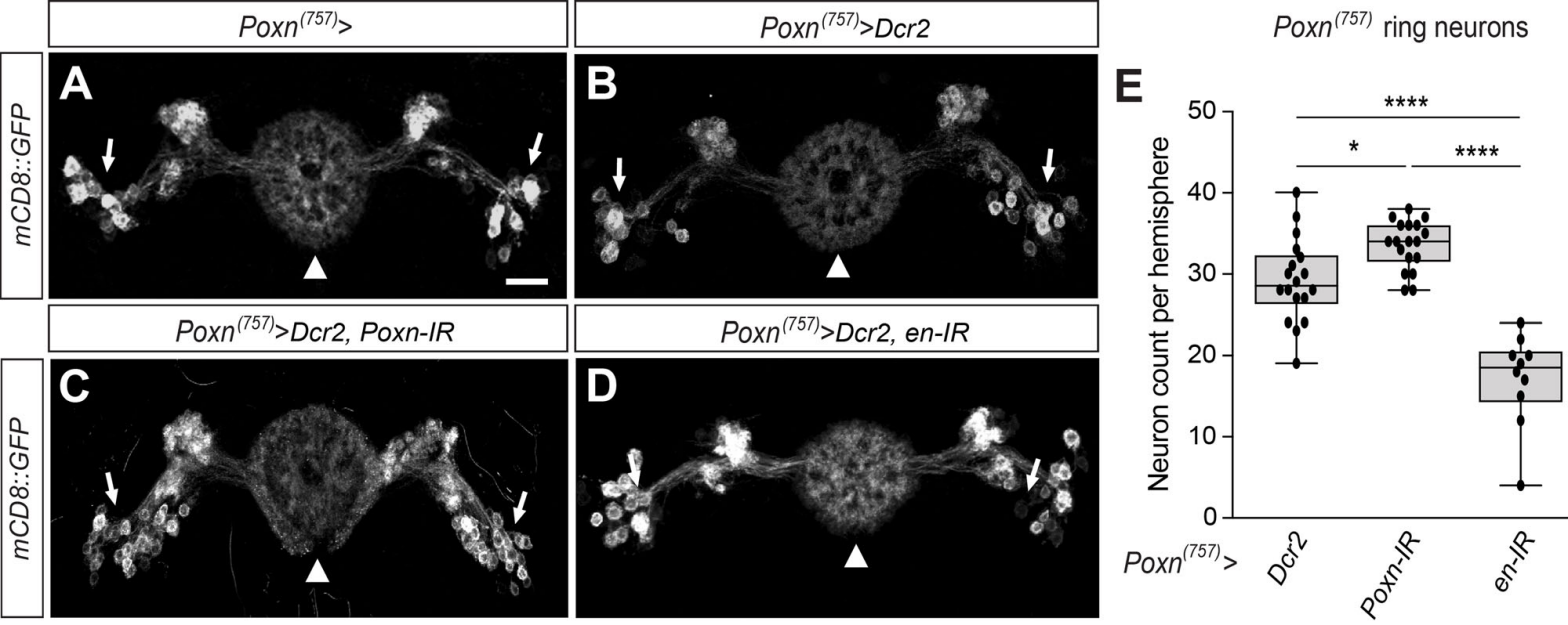
**Specification of EB ring neuron** **requires *Poxn* and *engrailed*.** Confocal images of whole-mount adult *Poxn^(757)^>mCD8::GFP* brains. Dorsal is up. (A) *Poxn^(757)^>mCD8::GFP* visualises selection of EB ring neurons (arrows) and their projections terminating in R1–R4 layers of the EB ring neuropil (arrowhead). (B) A comparable GFP expression pattern is seen in *Poxn^(757)^>mCD8::GFP, Dcr2* brains. (C) In *Poxn^(757)^>mCD8::GFP, Dcr2, Poxn-IR* brains*,* RNAi-mediated knockdown of *Poxn* reveals more GFP-positive EB ring neurons (arrows) and a ventrally open EB neuropil (arrowhead). (D) In *Poxn^(757)^>mCD8::GFP, Dcr2, en-IR* brains*,* RNAi-mediated knockdown of *engrailed* reveals less GFP-positive EB ring neurons (arrows) and a diminished outer layer of the EB ring neuropil (arrowhead). (E) Quantification of *Poxn^(757)^>mCD8::GFP* targeted EB ring neurons reveals increased cell numbers in *Poxn^(757)^>mCD8::GFP, Dcr2, Poxn-IR* brains (*n*=18, mean=33, s.e.m.=0.71, *P=0.0186) compared to *Poxn^(757)^>mCD8::GFP, Dcr2* controls (*n*=18; mean=29, s.e.m.=1.21), but significantly less cells in *Poxn^(757)^>mCD8::GFP, Dcr2, en-IR* (*n*=10; mean=17, s.e.m.=1.81, ****P<0.0001). *P*-values were determined with Bonferroni's multiple comparisons test, see Table S1 for details. Scale bar: 10 μm.

The observed ventrally-open EB ring phenotype in *en>mCD8::GFP,Dcr2,poxn-IR* brains suggested that ppd5-derived progeny devoid of *Poxn* may not adopt a proper ring-neuron identity. To test this hypothesis, we carried out experiments utilising a brain-specific *Poxn-Gal4* driver we generated, *Poxn^(757)^*, that shows activity in only a subset of Poxn-expressing ring neurons (Fig. 6A), thus allowing for analysis at single-cell resolution. To potentiate RNAi-mediated knockdown we again co-expressed Dcr2. Similar to *en>mCD8::GFP,Dcr2* brains, analysis of *Poxn-Gal4^(757)^>mCD8::GFP,Dcr2* brains revealed GFP-labelled EB ring-neuron morphology and projections into the ring neuropil (Fig. 6B). We then analysed GFP-labelled ring neurons targeted by RNAi-mediated knockdown of *Poxn* which identified a ventrally-open EB ring neuropil in *Poxn^(757)^>mCD8::GFP,Dcr2,Poxn-IR* brains (Fig. 6C) comparable to *en>mCD8::GFP,Dcr2,poxn-IR* (Fig. S7G–L). Moreover, the number of GFP-labelled ring neurons (Fig. 6E and Table S1) in *Poxn^(757)^>mCD8::GFP,Dcr2,Poxn-IR* brains was significantly increased (mean=33, s.e.m.=0.71; *n*=18) compared to *Poxn^(757)^>mCD8::GFP,Dcr2* controls (mean=29, s.e.m.=1.21; *n*=18). These data suggest a lineage-specific requirement for *Poxn* to specify the number and identity of EB ring neurons.

The observed phenotypes indicate a rather late function of *Poxn* in EB ring-neuron specification and we wondered whether *engrailed* might also have a later function in addition to its early requirement for embryonic-lineage formation. We therefore carried out *Poxn-Gal4^(757)^*-driven RNAi-mediated knockdown of *engrailed* which identified typical *Poxn^(757)^>mCD8::GFP*-labelled ring neurons and their projections into the EB ring neuropil, as shown for *Poxn^(757)^>mCD8::GFP,Dcr2,en-IR* brains (Fig. 6D). However, the *Poxn^(757)^>mCD8::GFP,Dcr2,en-IR*-labelled EB ring neuropil was lacking the outer synapse layer typical for R2/4 neurons (Fig. 6D, compare to A,B). Moreover, the number of *Poxn^(757)^>mCD8::GFP*-labelled ring neurons (Fig. 6E and Table S1) in *Poxn^(757)^>mCD8::GFP,Dcr2,en-IR* brains was significantly reduced (mean=17; s.e.m.=1.81; *n*=10). Together these data demonstrate that *engrailed* functions in *Poxn^(757)^-Gal4*-targeted cells and is required for the specification of the number and identity of R2/4 neurons to establish the outer layer of the EB ring neuropil.

## DISCUSSION

### Lineage-specific formation of EB ring-neuron circuitry

Previous studies suggested the *Drosophila* EB – as part of the central complex – develops from precursor cells that differentiate during larval development and during pupal stages generate the EB neuropil (Hanesch et al., 1989; Renn et al., 1999; Ito and Awasaki, 2008; Yu et al., 2009a,b; Bayraktar et al., 2010; Young and Armstrong, 2010; Omoto et al., 2017). Our lineage analysis demonstrates that at least part of its origin can be traced back to the embryonic procephalic neuroectoderm. We identified Engrailed-expressing neuroblasts ppd5 as embryonic stem cells that give rise to Poxn-expressing progeny, which ultimately differentiate into EB ring neurons. Genetic tracing with *en-Gal4* identified R1–R4 ring neurons, suggesting that embryonic neuroblasts ppd5 are the major source of Poxn-expressing progeny leading to EB ring neurons detected in our study. Based on their position, morphology, gene expression patterns and axonal fasciculation, our findings suggest that ppd5-derived larval lineages (Fig. 3) correspond to previously described larval lineages variously called ‘EB-A1/P1’ (Ito and Awasaki, 2008; Ito et al., 2013; Yu et al., 2013; Yang et al., 2013), ‘DALv2/3’ (Spindler and Hartenstein, 2011; Lovick et al., 2013; Omoto et al., 2017), ‘MC1’ (Kumar et al., 2009) or ‘DM’ (Bayraktar and Doe, 2013; Yang et al., 2013). We previously demonstrated that these larval lineages express Poxn and give rise to gamma-amino butyric acid (GABA)-ergic ring neurons in the central complex of the adult brain (Shaw et al., 2018). We therefore propose to (re-) name them according to their embryonic origin.

Subclass-specific Gal4 lines together with Poxn expression identifies these lineage-related, ppd5-derived sister cells as R1–R4 ring neurons. Moreover, brain-specific *Poxn-Gal4* mediated labelling identifies ring neurons and their axonal projections covering all layers of the EB neuropil, thus suggesting neuroblasts ppd5 give rise to the majority, if not all, of ring neuron subtypes. The ontogenetic relationship between Engrailed-expressing neuroblasts ppd5 and Poxn-expressing EB ring neurons is affirmed by the fact that *en-Gal4* and *Poxn-Gal4-*targeted RNAi-mediated knockdown of *Poxn* causes similar EB neuropil-specific phenotypes. Together, these data establish that ppd5-derived progeny are clonal units contributing to the EB ring neuron circuitry in the central complex in *Drosophila*.

### Lineage-related *Poxn* and *engrailed* function specifies EB ring neurons

How are these units specified? In both insects and mammals, the patterning and specification of neural lineages is regulated by genetic programs from neurogenesis to neuronal differentiation (e.g. Skeath and Thor, 2003; Guillemot, 2005; Gao et al., 2013; Allan and Thor, 2015). Our study in *Drosophila* shows that the development and specification of EB-specific circuit elements is likewise dependent on the lineage-specific activity of developmental regulatory genes. Early formation and maintenance of Poxn-expressing ppd5 lineages requires *engrailed* function as revealed with a deficiency removing both *engrailed* orthologues, *en* and *invected* (Fig. 2H,I). Previous studies showed that, *engrailed*/*invected* are required for the specification of neuroblast identity in the developing nervous system (Bhat and Schedl, 1997), suggesting that *engrailed* is also required for the specification of ppd5. We also found a later, lineage-specific function of *engrailed* in the specification of ring neuron numbers (Fig. 6), which is consistent with its transient expression in Poxn+ lineages in the embryonic brain (Fig. 3A,B) but not at later developmental stages nor in adult ring neurons (Figs 3M and 4A–E). *engrailed* codes for a homeodomain transcription factor mediating the activation and suppression of target genes, regulatory interactions that are required for neural lineage formation and specification in the procephalic neuroectoderm (McDonald and Doe, 1997; Gallitano-Mendel and Finkelstein, 1997; Seibert and Urbach, 2010). In contrast, no function for *Poxn* in embryonic brain development has been reported (Awasaki and Kimura, 1997, 2001; Boll and Noll, 2002; Minocha et al., 2017), suggesting that Poxn is only during later stages of development required for lineage and/or neuronal specification in the central brain.

Indeed, our experiments identify a postembryonic requirement of *Poxn* in the specification of ppd5-derived progeny. Previous studies showed that zygotic mutations of *Poxn* perturb EB neuropil formation, in that presumptive ring neurons are unable to project their axons across the midline and as a consequence, the EB ring neuropil is not formed (Boll and Noll, 2002; Minocha et al., 2017). In the present study, *en-Gal4*-targeted knockdown of *Poxn* reveals Engrailed-expressing cells that project across the midline and form a ring-like neuropil instead of their normal ipsilateral projections to the SP. Significantly, we did not observe any ppd5-derived GFP-labelled cells that project ipsilaterally towards the SP, neurons that are normally detectable with *en-Gal4* targeted GFP expression in the adult brain (Fig. 5B, asterisks). Furthermore, *en>Poxn-IR*-targeted, EB neuron-like projections do not form a torroidal ring but are rather characterised by a ventral cleft. These *en>Poxn-IR* cells aberrantly retain Engrailed expression even though their axonal projection and connectivity pattern clearly identify them as ring neurons that are normally devoid of Engrailed but instead express Poxn (Fig. 4C–E). Together these data suggest that, based on their morphology, Engrailed expression, axogenesis and ring-specific projection patterns, *en>GFP* cells normally projecting to the SP have been transformed into EB ring neurons in *en>mCD8::GFP,Dcr2,Poxn-IR* flies.

**Fig. 7.**
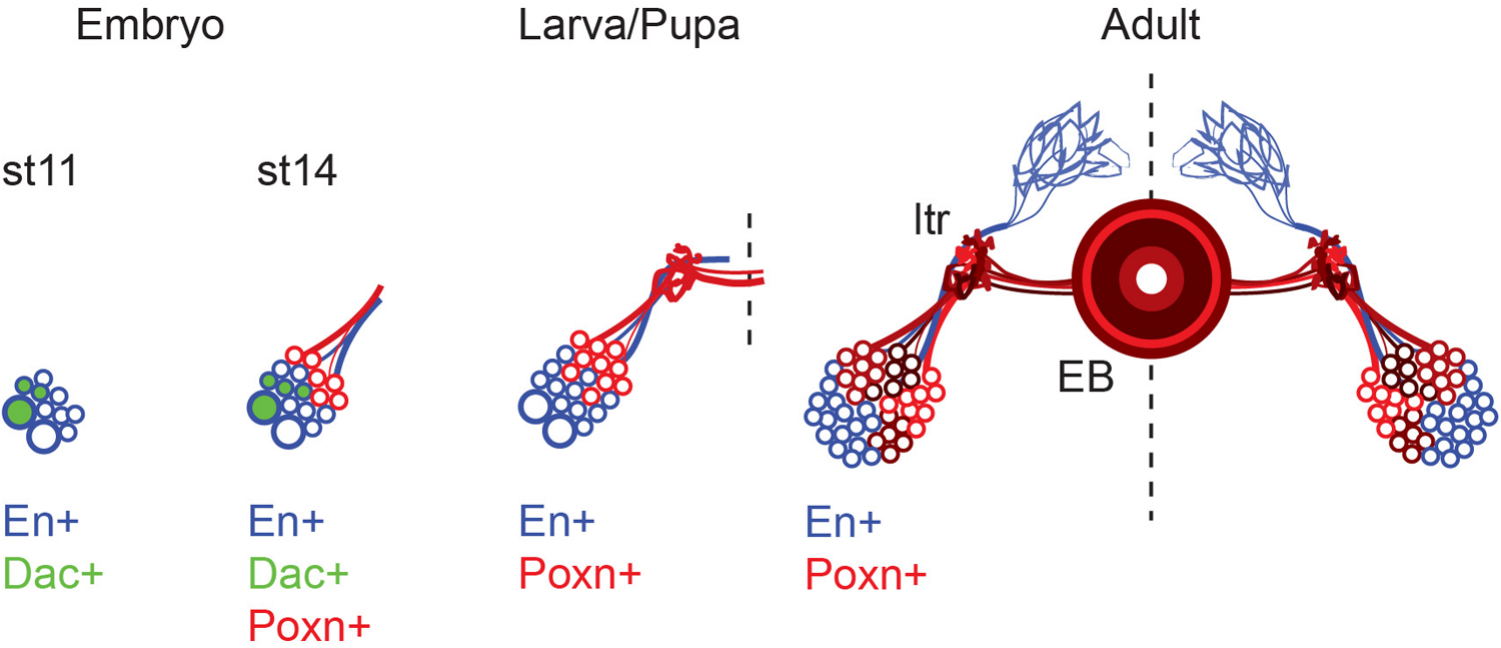
**Poxn-expressing EB ring neurons R1–R4 descend from *engrailed*-expressing neuroblasts ppd5.** During embryogenesis, *engrailed*-expressing neuroblasts ppd5 and ppd8 (large blue circles) derive from the procephalic neuroepithelium; they can be distinguished by Dachshund expression (Dac+) restricted to ppd8. At stage 11, ppd5/8 have produced a small number of Engrailed-expressing progeny (small blue circles). At stage 14, two classes of ppd5/8-derived neuron are visible: En^+^/Poxn^−^ (small blue circles) and En^−^/Poxn^+^ (small red circles). At this stage, cells are already sending axons towards the interhemispheric commissure. The lineages continue to expand during larval and pupal development and acquire their adult morphology during metamorphosis. Genetic tracing and mosaic analysis with a repressible cell marker identify En^+^/Poxn^−^ (small blue circles) and En^−^/Poxn^+^ (small red circles) as hemi-lineages derived from bilateral symmetric neuroblasts ppd5. Poxn neuro expression identifies R1–R4 ring neurons of the adult EB.

The resulting additional ring neurons in *en>mCD8::GFP,Dcr2,Poxn-IR* flies are accompanied with a ventrally open EB ring neuropil. A comparable phenotype is seen in brains of *Poxn^(757)^>Poxn-IR* flies which are characterised by an increased number of *Poxn^(757)^-Gal4*-targeted ring neurons, suggesting that increasing numbers of EB ring neurons lead to an arch-like neuropil reminiscent of the arch-like EB seen in the majority of arthropods (Strausfeld, 2012). In support of this notion, we previously demonstrated that *in vivo* amplification of ppd5-derived progenitor cells can lead to fully differentiated supernumerary GABAergic ring neurons that form functional connections often characterised by a ventrally open EB ring neuropil (Shaw et al., 2018). Together, these data identify differential roles of Poxn activity during neuroblast lineage formation, in that Poxn is required for cell identity determination of ppd5-derived progeny, as well as for the specification of cell numbers and terminal neuronal projections of EB ring neurons (Fig. 7).

These Poxn functions in ppd5-derived brain lineages are reminiscent of *Poxn* activity in the peripheral nervous system (PNS) which mediates the specification of sensory organ precursor (SOP) cell lineages giving rise to external sense organs, the tactile and gustatory bristles, respectively (Ghysen and Dambly-Chaudière, 2000). In these SOP lineages, differential *Poxn* activity determines progeny fate between chemosensory (gustatory) or mechanosensory (tactile) neuronal identities (Dambly-Chaudière et al., 1992; Awasaki and Kimura, 1997; Layalle et al., 2004). Furthermore, SOP lineage-specific *Poxn* function specifies the number of these neurons and their connectivity pattern (Nottebohm et al., 1992, 1994; Awasaki and Kimura, 2001). The apparent functional commonalities between *Poxn*-mediated specification of ppd5 neuroblast-derived lineages in the brain and SOP lineages in the PNS, suggest that evolutionarily-conserved mechanisms (Alberch, 1991; Hirth and Reichert, 1999) underlie the development and specification of clonal units as cellular substrates for neural circuit and sensory organ formation.

### Clonal units as cellular substrates for neural circuit evolution

The cytoarchitecture of both the insect and mammalian brain are characterised by neural lineages generated during development by repeated asymmetric divisions of neural stem and progenitor cells (Shen et al., 1998; Kim and Hirth, 2009; Sousa-Nunes and Hirth, 2016). These ontogenetic clones are thought to constitute building blocks of the insect and mammalian brain (Ito and Awasaki, 2008; Rakic, 2009). In support of this notion, lineage-related progeny constitutes sets of circuit elements of the mushroom bodies (Ito et al., 1997) and antennal lobes in *Drosophila* (Lai et al., 2008). Clonal relationship also characterises the lineage-dependent circuit assembly in the mammalian brain, where stem cell-like radial glia give rise to clonally-related neurons that synapse onto each other, as has been shown for cortical columns and GABAergic interneurons in the neocortex (Noctor et al., 2001; Yu et al., 2009a,b; Brown et al., 2011; Xu et al., 2014; Shi et al., 2017) and for striatal compartments of the basal ganglia (Kelly et al., 2018). Our study in *Drosophila* shows that a pair of bilateral symmetric, engrailed-expressing embryonic stem cells, neuroblasts ppd5, give rise to R1–R4 subtypes of tangential ring neurons that contribute to the layered EB neuropil. Thus, ppd5 neuroblast lineages constitute complete sets of circuit elements intrinsic to the adult central complex in *Drosophila* (Fig. 7).

It has been suggested that clonal expansion of neural lineages contributed to the evolution of complex brains and behaviours (Fish et al., 2008; Enard, 2011; Nielsen, 2015). Key to this hypothetical scenario are ancestral circuit elements in the form of genetically encoded stem cell-derived clonal units, like the ones described in our study here. In such a scenario, lineage-related ancestral circuit elements might have been multiplied and co-opted or diversified during the course of evolution. Multiplication and co-option have been suggested for the evolution of the multiple-loop architecture of the basal ganglia that allows processing of cognitive, emotional and motor information (Stephenson-Jones et al., 2011; Enard, 2011). In line with this hypothesis, quantitative control of the transcription factor *Prospero* is sufficient to cause clonal expansion of ring-neuron circuitry in *Drosophila* (Shaw et al., 2018), which has been implicated in cognitive and motor information processing (e.g. Fiore et al., 2015; Fiore et al., 2017; Kottler et al., 2019) and resembles extensive correspondences to vertebrate basal ganglia, ranging from comparable developmental genetics to behavioural manifestations and disease-related dysfunctions (Strausfeld and Hirth, 2013).

In contrast to multiplication and co-option, the diversification of stem cell lineages can equally contribute to neural circuit evolution. Our results presented here identify differential and tightly regulated spatio-temporal functions of *engrailed* and *Poxn* that lead to the differentiation of ppd5 progeny into hemi-lineage specific identities in the adult brain. Loss of *engrailed* affects the formation of precursors cells, whereas its lineage-specific knockdown affects the number of Poxn expressing ring neurons. Correspondingly, en-Gal4-driven lineage-specific knockdown of *Poxn* results in an identity transformation of Engrailed-expressing neurons in the adult brain in that they no longer project to the SP, but instead reveal an EB ring-neuron identity. These data indicate a binary switch of hemi-lineage identities as the result of a feed-forward mechanism between *engrailed* and *Poxn. engrailed* may activate transcription (directly or indirectly) of *Poxn,* which in turn represses *engrailed* to permit differentiation of R1–R4 neurons, thereby regulating the specification of neuronal identities in ppd5 hemi-lineages. This hypothesis is consistent with lineage tracing (Fig. 4) and MARCM experiments (Fig. S5), as well as the transient expression of *engrailed* in embryonic ppd5 lineages but not in adult EB ring neurons. However, further studies are required to elucidate the nature and extend of these putative regulatory interactions between *Engrailed* and *Poxn*.

In summary, our findings presented here establish a causal relationship between a pair of bilateral symmetric embryonic stem cells, neuroblasts ppd5 and the lineage-related assembly of their EB ring neuron progeny as structural units of the central complex in *Drosophila*. Based on these observations we propose that amplification and diversification of ontogenetic clones together with the repurposed use or exaptation (Gould and Vrba, 1982) of resulting circuitries, is a likely mechanism for the evolution of complex brains and behaviours.

## MATERIALS AND METHODS

### Drosophila genetics

All lines were obtained from the Bloomington Stock Center and raised at 25°C with a 12 h/12 h light/dark cycle. Embryonic and larval gene expression studies were carried out using *w^1118^; +; +* and *w; en-Gal4, UAS-mCD8::GFP/(CyO); +* (*en>mCD8::GFP*), unless otherwise stated.

To generate *Poxn^brain^-Gal4* flies, the *Poxn* brain enhancer (Boll and Noll, 2002) was amplified by PCR from genomic DNA. The PCR product was sub-cloned into *pPTGal* vector using *XbaI* and *NotI* sites, followed by sequencing; the genomic region 2R:11723830 to 11725559 was inserted into *pPTGal*. Primer sequences are: forward, *5'-gctcattaatgaccatgaaa-3′*; reverse, *5'-aagcggccgcgttaagtaacgctcggtgg-3′*. Transgenesis was performed by BestGene Inc (CA, USA).

For lineage tracing, the following strains were used: *w^1118^* (control), *en-Gal4* (*en>mCD8::GFP*), *Poxn-Gal4* or *Dac-Gal4* were crossed to *UAS-mCD8::GFP, tub-FRT-CD2-FRT-Gal4,UAS-FLP/CyO GMR Dfd YFP* (Roy et al., 2007). Offspring were raised at 18°C to suppress leaky or unspecific FLP activity.

For analysis of ring-neuron subtypes, the following enhancer trap lines were used: *c42-Gal4*, *c105-Gal4*, *c507-Gal4* and *c819-Gal4* (Renn et al., 1999; from S. Goodwin, University of Oxford), *EB1-Gal4* (Wang et al., 2002; from T. Lee, HHMI Janelia Research Farm), *c232-Gal4* (Renn et al., 1999; from J. R. Martin, Paris-Saclay Institute of Neuroscience), as well as *796-Gal4* (Martín-Peña et al., 2006; from A. Ferrus, Cajal Institute Madrid) in combination with *yw; P{UASmCD8::GFP.L}LL5; +*.

To study specification of ring-neuron precursors, the *engrailed* deficiency *f^36a^; Df(2R)en^E^, en^E^, inv^E^/CyO; mwh^1^, jv^1^, P{f^+13^}77A/TM2* was used. RNAi was carried out using *en>mCD8::GFP* or *Poxn^(757)^-Gal4*. The lines *UAS-Dcr2*, *UAS-Poxn-RNAi*, and *UAS-en-RNAi* were obtained from the Vienna Drosophila RNAi Centre (Dietzl et al., 2007) and the Bloomington Stock Center; experimental strains carrying *Dcr2* and each RNAi construct were generated by genetic crosses using the double balancer line *w; If/CyO; MKRS/TM6b,Tb,Hu*. For overexpression of engrailed, we made use of *UAS-engrailed* (*y w hs.FLP122; UAS.en/ TM2;* from J. Casal, University of Cambridge, UK). For overexpression of Poxn, we generated transgenic *UAS-Poxn* lines using the full-length open reading frame of Poxn (*Poxn* cDNA clone IP01592, Berkeley Drosophila Genome Project from the Drosophila Genomics Resource Center). cDNA was 6× His tagged at the N-terminus and sub-cloned into *pUAST*. Transgenesis was performed by BestGene Inc (CA, USA).

### Immunohistochemistry and image analysis

Immunostainings were performed as previously described (Hirth et al., 2003; Diaper et al., 2013; Diaper and Hirth, 2014). Rabbit anti-Poxn antibody was generated using *pUAST-HisPoxn*-derived protein purified by GenScript (New Jersey, USA). Purified Poxn protein was injected into rabbits for antibody production by Pab productions (Hebertshausen, Germany).

Primary antibodies used were: mouse anti-Dachshund, 1:20 (mAbdac2-3, Developmental Studies Hybridoma Bank, DSHB); mouse anti-Engrailed, 1:2 (4D9, DSHB); rabbit and chicken anti-GFP, 1:500 (Thermo Fisher Scientific/Invitrogen, A6455 and Ab13970, Abcam, respectively); goat anti-HRP (Cy3 conjugated- 123-165-021, Cy5 conjugated–115-175-146), 1:50 (all Jackson ImmunoResearch Labs); rabbit anti-Poxn, 1:200 (Boll and Noll, 2002; from M. Noll, University of Zurich); mouse anti-Poxn, 1:100 (Hassanzadeh, et al., 1998; a kind gift from A. Ghysen, University of Montpellier); rabbit anti-Poxn, 1:400 (generated as described above); mouse anti-Synapsin, 1:50 (3C11, DSHB); mouse anti-Brp, 1:20 (nc82, DSHB); rabbit anti-Miranda, 1:200 (Shaw et al., 2018); mouse anti-Prospero, 1:5 (mAbMR1A, DSHB); rabbit anti-pH3, 1:400 (06-570, Sigma-Aldrich);. Secondary antibodies were Alexa fluorochromes at 1:150 (Invitrogen). Embryos, larval CNSs and adult brains were mounted in Vectashield with DAPI (H-1200, Vector Laboratories).

Fluorescence samples were scanned and recorded either with a Leica TCS SP5 or A1R Nikon confocal microscopes in sequential scanning mode. Leica TCS SP5 was equipped with Leica Application Suite Advanced Fluorescence (LAS-AF) software, HCX PL APO lambda blue 20.0×0.70 IMM UV 0.70 numerical aperture (NA) and HCX PL APO CS 40.0×1.25 OIL UV 1.25 NA objectives. A1R Nikon confocal was equipped with Elements Confocal software, Plan Fluor 40× oil DIC H N2 NA 1.3 and Plan Apo VC 20× DIC N2 0.75 NA objectives. Whole-mount adult brains were scanned using the same confocal settings for each genotype. Z-projections were created and analysed using FIJI. Neurons expressing *UAS-mCD8::GFP* were counted using the ImageJ Cell Counter Plugin (http://rsbweb.nih.gov/ij/plugins/cell-counter.html). Images were processed using Adobe Photoshop and figures constructed in Adobe Illustrator.

### Statistics

Statistical analysis was carried out using GraphPad prism 6. Comparison of means from multiple experimental conditions (>2) with one independent variable was performed using the one-way analysis of variance (ANOVA), followed by Bonferroni's multiple comparisons post-hoc test. The alpha level for all tests was 0.05, for details see Table S1.

## Supplementary Material

Supplementary information
