# Supplementary material for: Lineage-specific determination of ring neuron circuitry in the central complex of Drosophila
Source: Biol Open. 2019 Jul 8;8(7):bio045062. doi: 10.1242/bio.045062 (PMC6679397; doi:10.1242/bio.045062)
Supplement: Supplementary information [file biolopen-8-045062-s1.pdf]

SUPPLEMENTARY INFORMATION

Lineage-specific determination of ring neuron circuitry in the central complex of *Drosophila*

Jessika C. Bridi[1,#], Zoe N. Ludlow[1,#] and Frank Hirth[1,*]

[1]Department of Basic and Clinical Neuroscience, Maurice Wohl Clinical Neuroscience Institute, Institute of Psychiatry, Psychology and Neuroscience, King's College London, United Kingdom.

[#]These authors contributed equally.

Correspondence to: Dr. Frank Hirth, King's College London, Institute of Psychiatry, Psychology and Neuroscience, Maurice Wohl Clinical Neuroscience Institute, Cutcombe Road, SE5 9RX, London, United Kingdom; Tel: ++44 20 7848 0786; email: Frank.Hirth@kcl.ac.uk

Biology Open • Supplementary information

Table S1. Quantification of phenotypes and statistical tests used.

	>Dcr2 ctrl	***>Poxn-IR***	***>en-IR***
Number of values	18	18	10
Minimum	19	28	4
25% Percentile	26.25	31.5	14.25
Median	28.5	34	18.5
75% Percentile	32.25	36	20.5
Maximum	40	38	24
Mean	29.17	33.56	17.1
Std. Deviation	5.136	3.034	5.724
Std. Error of Mean	1.211	0.715	1.81
Lower 95% CI	26.61	32.05	13.01
Upper 95% CI	31.72	35.06	21.19
Bonferroni's multiple comparisons	Mean Diff.	95% CI of diff.	Adj. P Value
Dcr2 ctrl vs. *Poxn-IR*	-4.389	-8.188 to -0.5901	0.0186
Dcr2 ctrl vs. *en-IR*	12.07	7.572 to 16.56	<0.0001
Poxn-IR vs. *en-IR*	16.46	11.96 to 20.95	<0.0001

Biology Open • Supplementary information

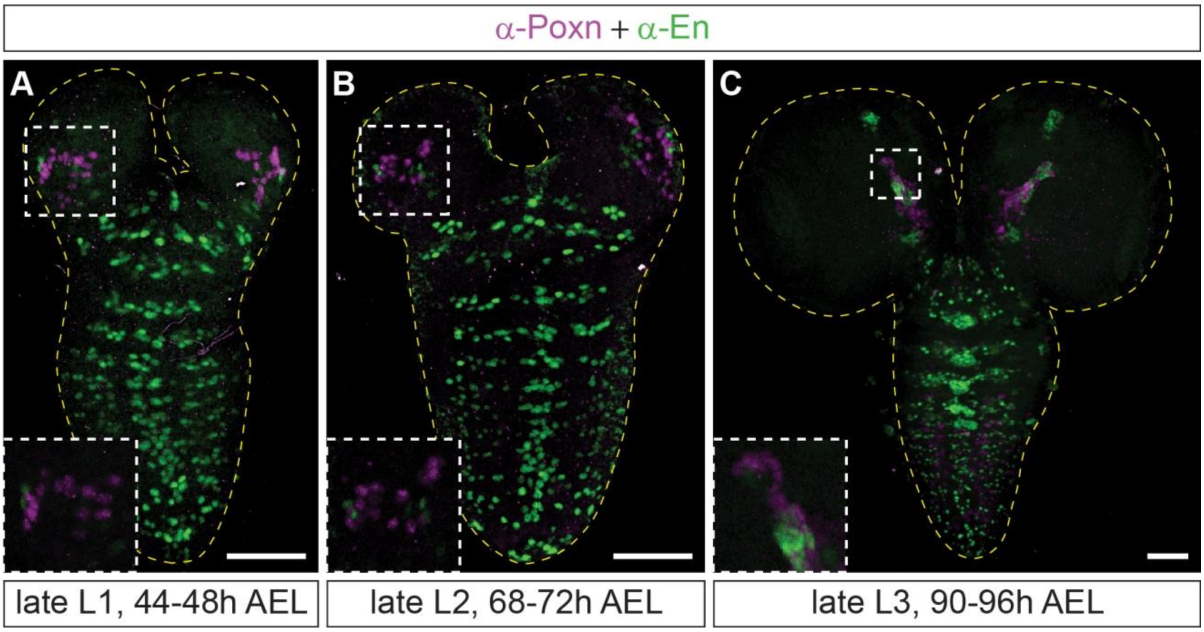

Fig. S1. Engrailed and Poxn expression in the larval central nervous system. Confocal images of whole-mount larval CNS' immunolabelled with anti-Poxn and anti-Engrailed; anterior is up. (**A**) Late L1 larval CNS, 44-48 hrs after egg laying (AEL). Poxn expression can be detected in two clusters of the anterior brain in close vicinity to En expressing cells, including the posterior protocerebrum (dashed squared area, enlarged bottom left). (**B**) Late L2 larval CNS, 68-72 hrs AEL. Poxn expression is still detectable in the posterior protocerebrum in close vicinity to En expressing cells (dashed squared area, enlarged bottom left). (**C**) The same applies to late L3 larval CNS, 90-96 hrs AEL. Poxn expression is still detectable in close vicinity to En expressing cells in the posterior protocerebrum (dashed squared area, enlarged bottom left). $n > 20$ for each condition. Scale bars: 50µm.

Biology Open • Supplementary information

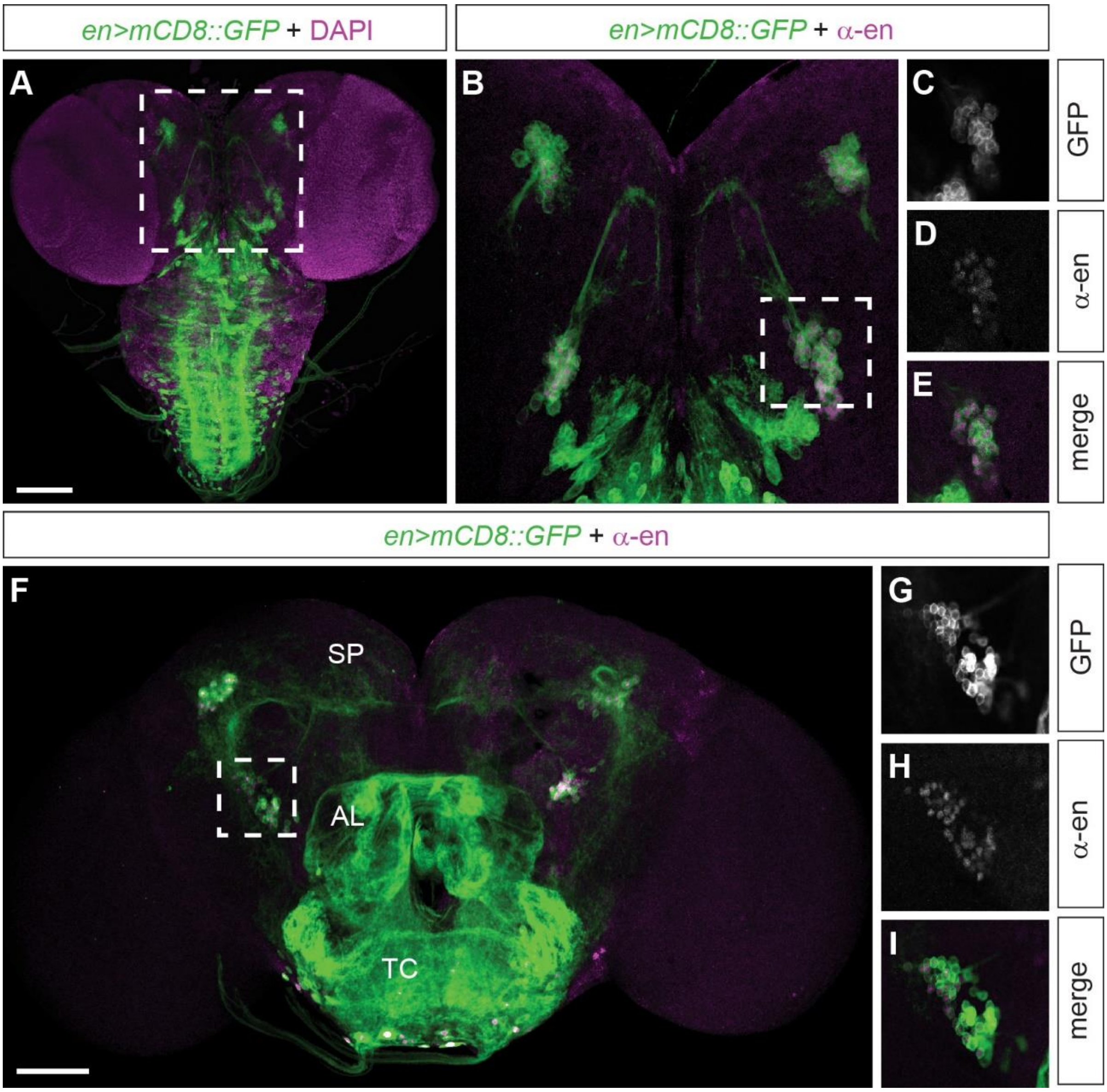

Fig. S2. *en-Gal4, UAS-mCD8::GFP* recapitulates endogenous Engrailed expression in L3 and adult brains. (**A**) late 3rd instar larval CNS expressing *en>mCD8::GFP* and co-labelled with DAPI to show the outline of the CNS. (**B**) enlargement of dashed area shown in A. Posterior protocerebral cluster of *en>mCD8::GFP*-expressing cells (dashed box) are also positive for anti-En. (**C**-**E**) enlargements of dashed area shown in B. There is complete colocalisation between *en>mCD8::GFP* and anti-En. (**F**) adult *en>mCD8::GFP* brain labelled with anti-En showing the adult *en>mCD8::GFP* projection pattern. (**G**-**I**) enlargements of dashed area shown in F. There is complete colocalisation between *en>mCD8::GFP* and anti-En. Abbrev.: SP, superior protocerebrum; AL, antennal lobes; TC, tritocerebrum. $n > 20$ for each condition; scale bars, 100μm.

Biology Open • Supplementary information

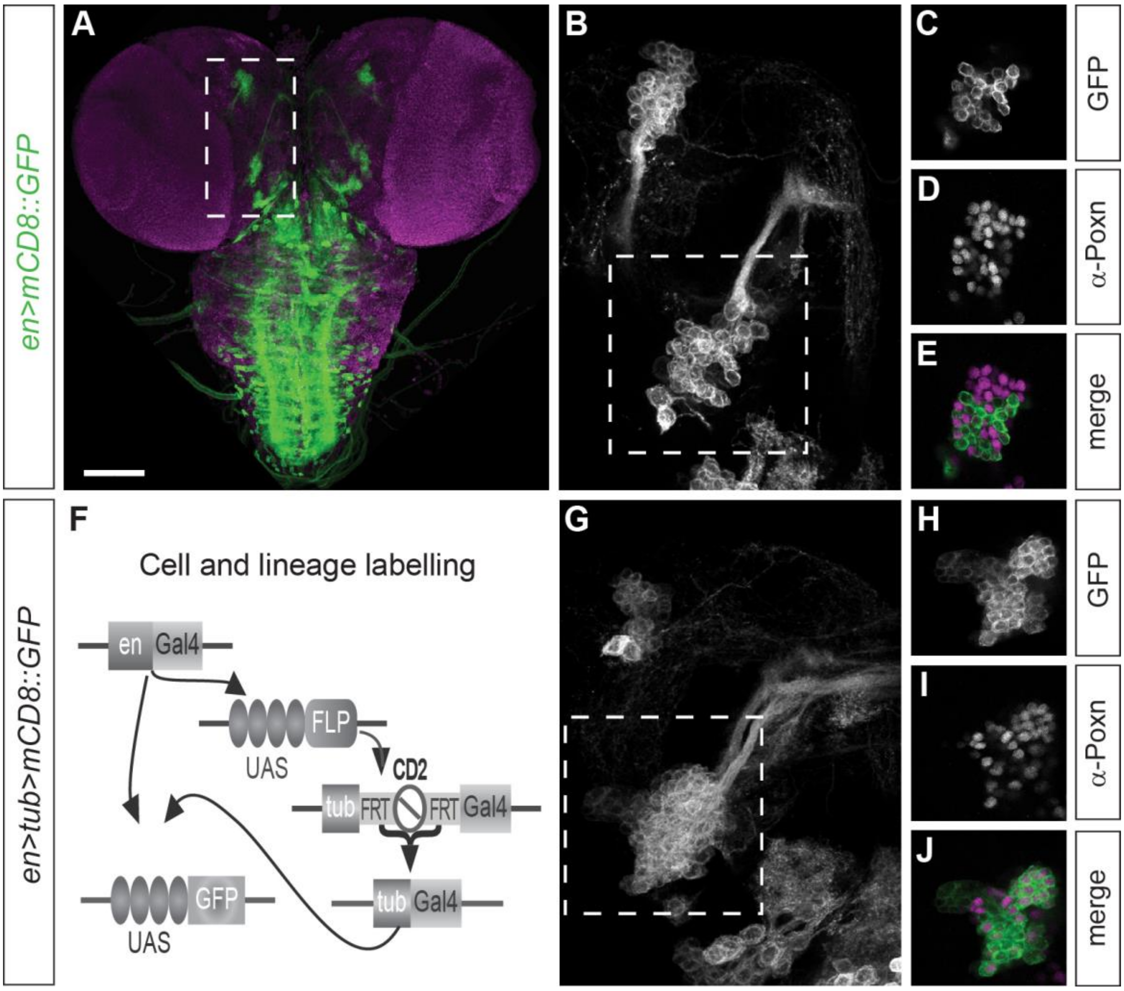

Fig. S3. Posterior protocerebral *engrailed* expressing cells visualised with *en>mCD8::GFP* and *en>tub>mCD8::GFP*. Confocal images of late 3rd instar larval CNS, anterior is up. (**A**) *en>mCD8::GFP* and DAPI labelling reveals engrailed expressing lineages in the larval CNS (same specimen as in Fig. S2A; dashed area illustrates enlarged area in B, but B shows different specimen). (**B**) *en>mCD8::GFP*-labelled cells in the posterior protocerebrum (dashed box) project anteriorly and towards the midline. (**C-E**) *en>mCD8::GFP*-labelled cells (C) in the posterior protocerebrum do not express Poxn (D, E). (**F**) schematic of cell and lineage labelling using *en>tub>mCD8::GFP*. *en-Gal4* activates expression of *UAS-FLP* which mediates mitotic recombination at FRT sites. This leads to excision of CD2 ‘stop’ element, allowing the *tub* enhancer to activate Gal4, which in turn maintains GFP expression in this cell and all its progeny. (**F**) Genetic tracing with *en>tub>mCD8::GFP* reveals more GFP-expressing cells than *en>mCD8::GFP* in the posterior protocerebrum (dashed box); neurons project anteriorly and towards the midline. (**G-J**) *en>tub>mCD8::GFP* labelling reveals GFP-expressing cells (G) that are immunolabelled with anti-Poxn (H-J). *n* > 20 for each condition. Scale bar: 100μm.

Biology Open • Supplementary information

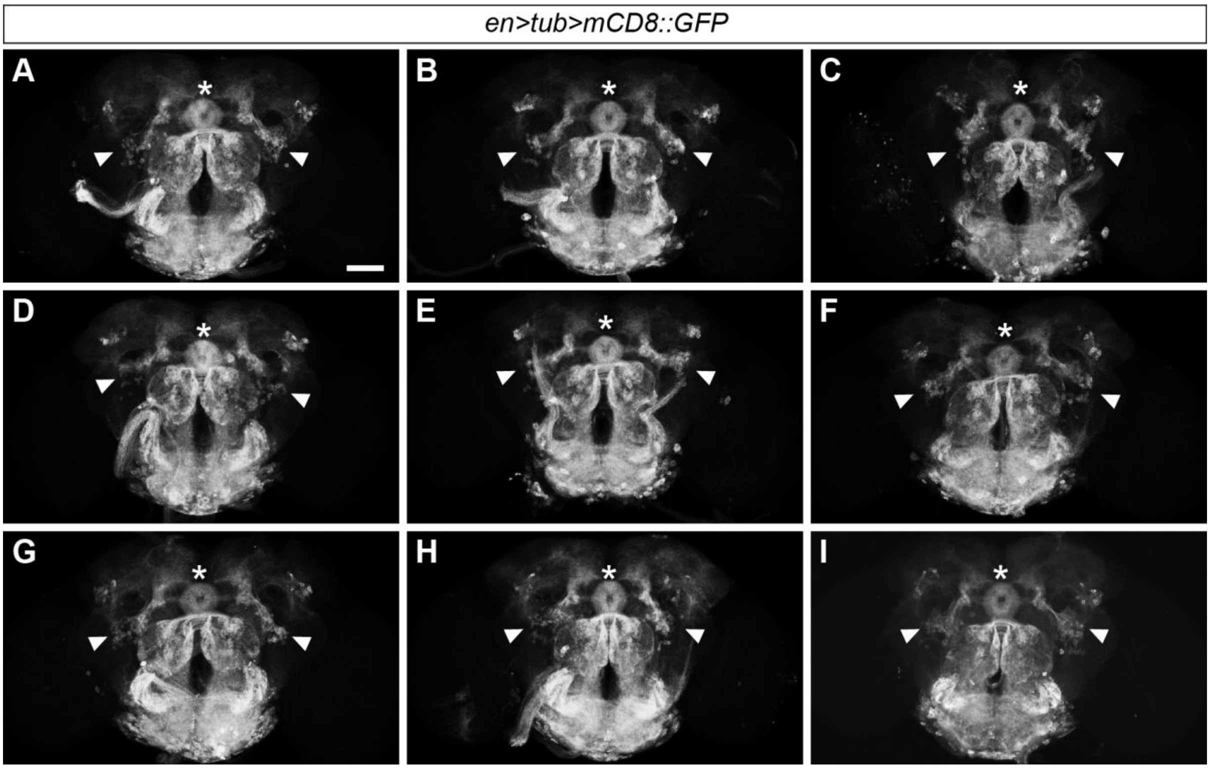

Fig. S4. Genetic tracing with *en>tub>mCD8::GFP* invariably labels the ellipsoid body in the adult brain. Confocal images of whole-mount adult brains; dorsal is up. (**A-I**) Genetic tracing with *en>tub>mCD8::GFP* consistently reveals GFP-expressing ellipsoid body (EB) ring neuron cell bodies (arrowheads) and their projections to the EB ring neuropil (asterisks). *n* = 70 brains. Scale bar: 100µm.

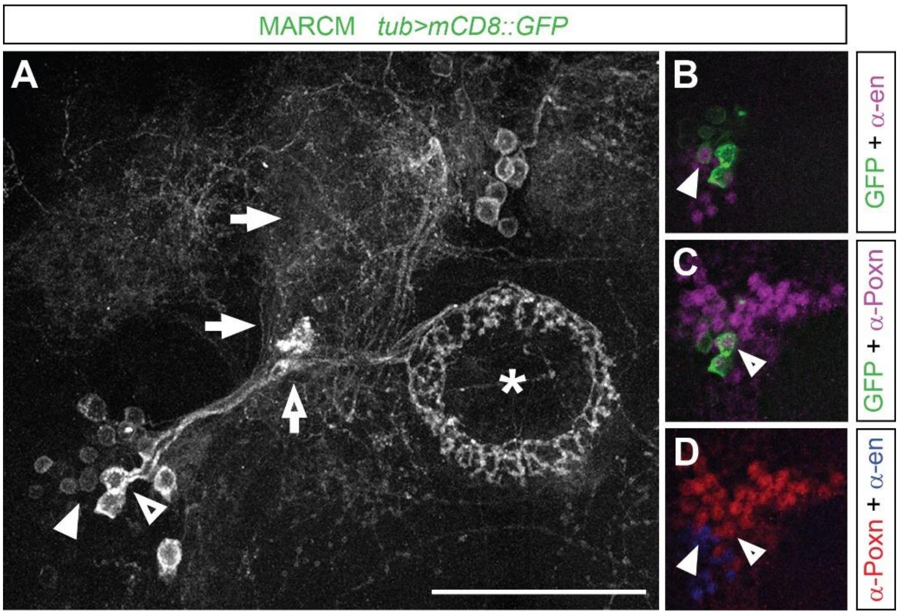

Fig. S5. Lineage analysis of Engrailed and Poxn-expressing neurons in the posterior protocerebrum. Mosaic analysis with a repressible cell marker (MARCM). (**A**) section of adult central brain showing a posterior protocerebral MARCM clone. The clone includes cells that express Engrailed (anti-en; solid arrowheads, see also B) and project to the superior protocerebrum (solid arrows); and cells expressing Poxn (antiPoxn; open arrowheads, see also C) that send projection to the ellipsoid body (open arrows) that terminate in the ring neuropil (asterisk). (**B**) anti-engrailed labels GFP-positive cells (arrowhead) of the MARCM clone. (**C**) anti-Poxn labels GFP-positive cells (arrowhead) of the MARCM clone. (**D**) Same section as B and C with co-immunolabelling of anti-Poxn adnd anti-en showing that Poxn and Engrailed expressing cell clusters are adjacent to one another. Note that the presence of both Poxn and Engrailed expressing cell types in a single MARCM clone demonstrates that their ontogenetic relationship as sister cells. $n > 20$. Scale bar: 50μm.

Biology Open • Supplementary information

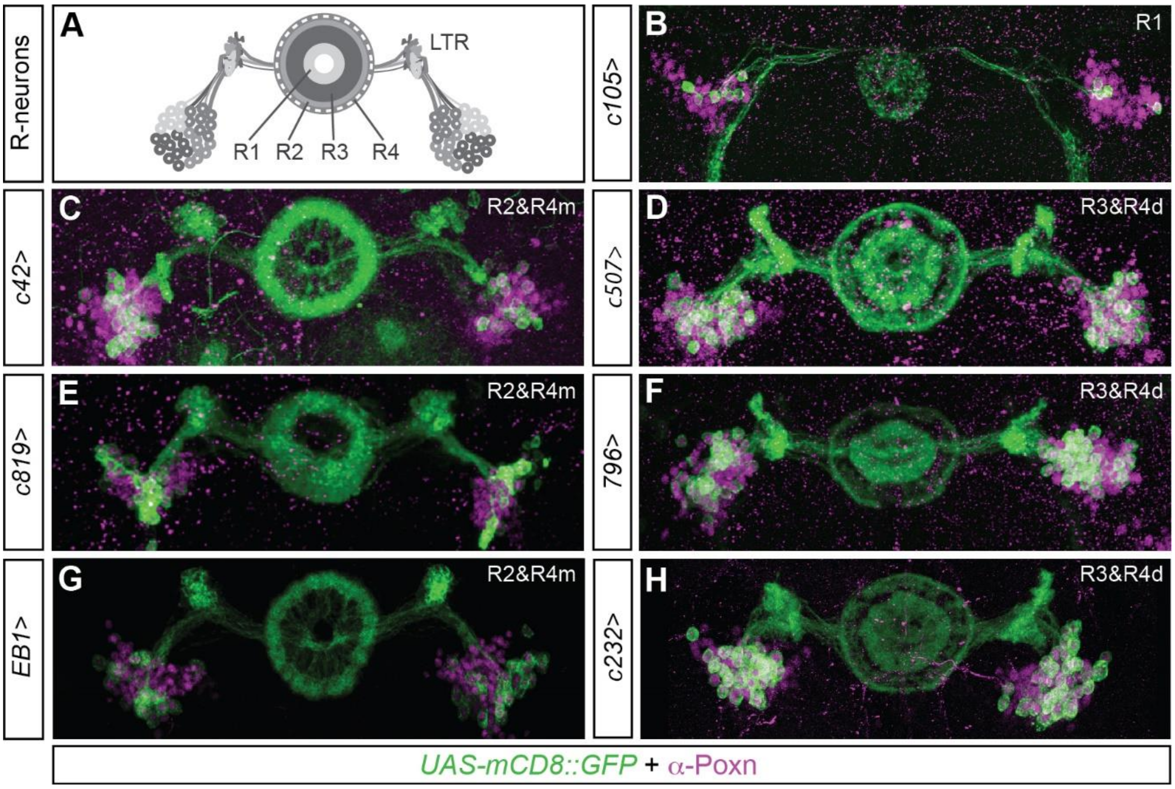

Fig. S6. Ellipsoid body ring neurons R1-R4 express Poxn. (**A**) Schematic of R1-R4 subtypes of ellipsoid body ring neurons and their layer-specific projections into the ring neuropil; LTR, lateral triangle. (**B**) *c105>mCD8::GFP* visualises R1 neurons. (**C**) *c42>mCD8::GFP* labels R2 & R4m neurons. (**D**) *c507>mCD8::GFP* visualises R3 and R4d neurons. (**E**) *c819>mCD8::GFP* visualises R2 & R4m neurons. (**F**) *796>mCD8::GFP* visualises R3 & R4d neurons. (**G**) *EB1>mCD8::GFP* visualises R2 & R4m neurons. (**H**) *c232>mCD8::GFP* visualises R3 & R4d neurons. Anti-Poxn immunolabelling (magenta) reveals Poxn expression in all subtypes of R1-R4 ellipsoid body ring neurons. n > 10 for each condition.

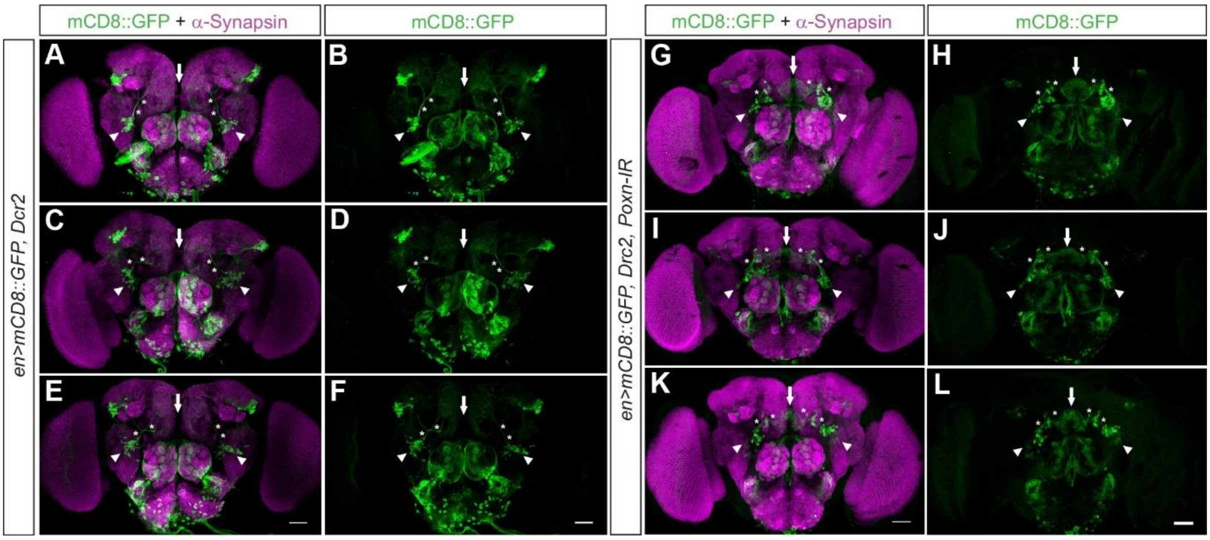

Fig. S7. *en*-Gal4 UAS-RNAi mediated knockdown of *Poxn*. Confocal images of whole-mount adult brains expressing *mCD8::GFP* and immunolabelled with anti-Syntaxin/3C11 (magenta); dorsal is up. (**A-F**) In *en>mCD8::GFP, Dcr2* brain, the ellipsoid body (EB) neuropil is not visualised (arrow); GFP expressing cells (arrowheads) send their projections ipsilaterally (asterisks) into the superior protocerebrum, dorsal to the EB ring neuropil (position indicated with arrow). (**G-L**) In *en>mCD8::GFP, Dcr2, Poxn-IR,* RNAi-mediated knockdown of *Poxn* in ppd5 lineages reveals GFP expressing cells (arrowheads) that send their projections contralaterally (asterisks) into the EB ring neuropil (arrow) which appears ventrally open to varying degrees. n > 10 for each condition. Scale bars: 50μm.

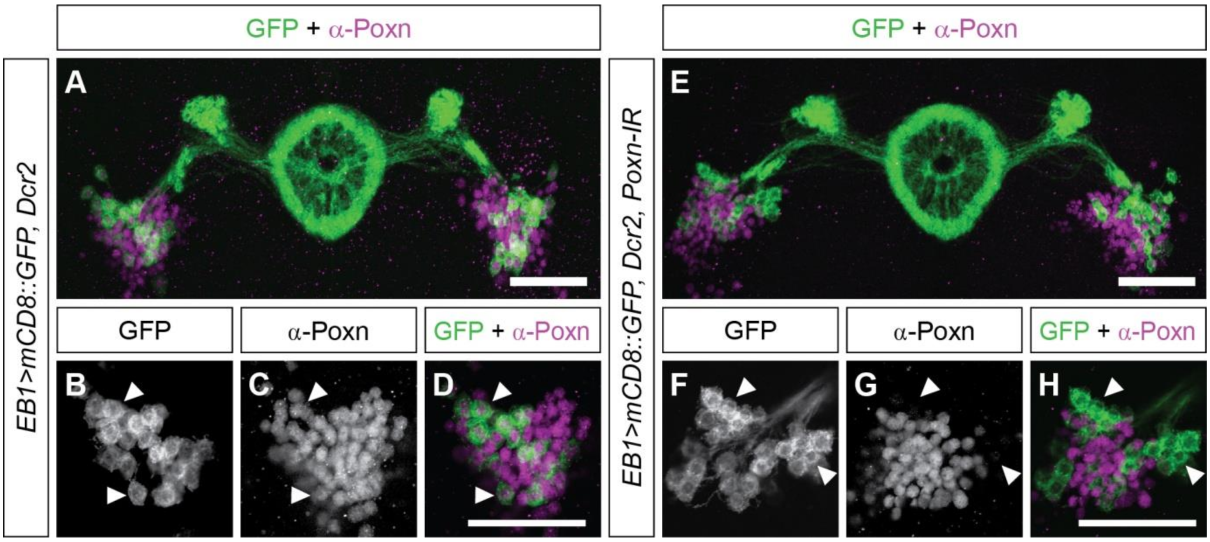

Fig. S8. RNAi-mediated knockdown of *Poxn* removes Poxn immunoreactivity. Confocal images of whole-mount adult brains immunolabelled with anti-Poxn (magenta); dorsal is up. (**A**) In *EB1>mCD8::GFP, Dcr2* brains, GFP-labelled ellipsoid body (EB) ring neurons send projections into the EB ring neuropil. (**B**) *EB1>mCD8::GFP, Dcr2,* cells express GFP and (**C**) are immunoreactive for anti-Poxn, revealing (**D**) that all GFP-positive cells are also labelled with anti-Poxn (arrowheads). (**E**) In *EB1>mCD8::GFP, Dcr2, Poxn-IR* brains, RNAi-mediated knockdown of *Poxn* reveals GFP-labelled cells in the posterior protocerebrum (arrowheads) sending projections to the EB (arrow). (**F**) *EB1>mCD8::GFP, Dcr2, Poxn-IR,* cells express GFP in close vicinity to (**G**) anti-Poxn immunoreactive cells; however (**H**) the majority of GFP expressing are devoid of anti-Poxn immunoreactivity (arrowheads). $n > 10$ for each condition. Scale bars: 50µm in A, E; 10µm in D, H.
